# Identification of novel circulating miRNAs biomarkers for healthy obese and lean children

**DOI:** 10.1186/s12902-023-01498-w

**Published:** 2023-10-30

**Authors:** Feifei Ma, Dingding Cao, Zhuo Liu, Yuanyuan Li, Shengrong Ouyang, Jianxin Wu

**Affiliations:** 1 Department of Biochemistry and Immunology, Capital Institute of Pediatrics, 2 Yabao street, Beijing, 100020 People’s Republic of China; 2 Institute of Basic Medical Sciences, School of Basic Medicine, Chinese Academy of Medical Sciences and Peking Union Medical College, 5 Dongdansantiao, Beijing, 100005 People’s Republic of China; 3 Beijing TongRen Hospital, Capital Medical University, 17 Hougou Street, Chong Wen Men, Beijing, 100730 People’s Republic of China

**Keywords:** Child, Simple obesity, microRNA, Target gene

## Abstract

**Background:**

The prevalence of childhood obesity and overweight has risen globally, leading to increased rates of metabolic disorders. Various factors, including genetic, epigenetic, and environmental influences such as diet and physical activity, contribute to pediatric obesity. This study aimed to identify specific circulating miRNAs as potential biomarkers for assessing obesity in children.

**Methods:**

Thirty children, including 15 obese and 15 extremely thin individuals, were selected for this study. MiRNA expression in circulating plasma was assessed using miRNA microarrays. The reliability of differential miRNA expression was confirmed using TaqMan qPCR. The correlation between miRNAs and obesity was analyzed through multiple linear regression, receiver operator characteristic (ROC) curve analysis, and odds ratio (OR) calculations. Bioinformatics tools were utilized to identify target genes for the selected miRNAs, and a functional network map was constructed.

**Results:**

A total of 36 differentially expressed miRNAs were identified through gene chip analysis, and TaqMan qPCR validation confirmed the upregulation of seven miRNAs: hsa-miR-126-3p, hsa-miR-15b-5p, hsa-miR-199a-3p, hsa-miR-20a-5p, hsa-miR-223-3p, hsa-miR-23a-3p, and hsa-miR-24-3p. Among these, hsa-miR-15b-5p and hsa-miR-223-3p exhibited a statistically significant difference except for hsa-miR-23a-3p. These two miRNAs showed more predicted target genes related to obesity than others. Multiple linear regression analysis revealed an association between obesity and hsa-miR-15b-5p and hsa-miR-223-3p [10.529 (4.974–16.084), -10.225 (-17.852~ -2.657)]. Even after adjusting for age and sex, these two miRNAs remained associated with obesity [8.936 (3.572–14.301), -8.449(-15.634~ -1.303)]. The area under the ROC curve (AUC) reached values of 0.816, 0.711, and 0.929, respectively. Odds ratio analysis demonstrated a significant correlation between obesity and hsa-miR-15b-5p (OR = 143, 95% CI 5.80 to 56,313, p = 0.024) and between obesity and hsa-miR-223-3p (OR = 0.01, 95% CI 0.00 to 0.23, p = 0.037). Importantly, hsa-miR-15b-5p was found to have numerous target genes associated with the FoxO, insulin, Ras, and AMPK signaling pathways.

**Conclusions:**

Differential miRNA expression profiles in the circulation of obese children compared to controls suggest underlying metabolic abnormalities. Hsa-miR-15b-5p and hsa-miR-223-3p may be considered as molecular markers for the screening of obese children and populations at risk of developing metabolic syndrome.

**Supplementary Information:**

The online version contains supplementary material available at 10.1186/s12902-023-01498-w.

## Background

The global incidence of obesity has witnessed a rapid and alarming increase. Notably, the prevalence of overweight and obesity among children and adolescents has surged [1, 2]. In tandem with China’s socioeconomic growth, improved living standards, and substantial shifts in family lifestyles, the issue of childhood obesity has become increasingly concerning. The third national epidemiological survey on childhood obesity, conducted in 2006, reported that over the past decade, the combined prevalence rates of obesity and overweight among children aged 0–6 years in China were 7.2% and 19.8%, respectively [3]. Recent years have witnessed a worrisome surge in the number of school-age children and adolescents affected by obesity and overweight. The incidence rates of obesity are on the rise among both urban and rural children. China is now home to 12 million overweight and obese children, constituting approximately one-thirteenth of the world’s “overweight children” population. This concerning trend signifies that adolescent obesity in China has entered an epidemic phase, posing a significant threat to the health and well-being of the Chinese population and emerging as a major public health challenge.

In recent years, advancements in life sciences and technology have led to an enhanced understanding of developmental processes and disease mechanisms. MicroRNAs (miRNAs) are endogenous, non-coding, single-stranded small RNA molecules, typically around 22 nucleotides in length. MiRNAs exert their regulatory function by imperfectly binding to target mRNAs, resulting in mRNA degradation or translational inhibition. MiRNAs are implicated in nearly all biological processes and contribute to the pathogenesis of numerous diseases [4]. This study aims to investigate circulating miRNA levels in children with simple obesity and low body weight, with the objective of exploring the potential of miRNAs as molecular markers for detecting obesity-related metabolic abnormalities in Chinese children.

## Methods

### Research object

For the circulating miRNA study, a total of 30 children (15 boys and 15 girls) aged 8–15 years who had undergone routine health physical examinations in primary and secondary schools in Beijing from August 2015 to April 2017 were selected. The body mass index (BMI) data for boys and girls in urban and rural areas, as reported in the “Investigation report on Chinese students’ physique and health in 2000” [5], was utilized to standardize the BMI values. BMI was calculated as weight (kg) divided by height squared (m^2^), and the fat mass percentage (FMP) was determined through the bioelectrical impedance method. The participants were categorized into two groups: the obesity group (comprising individuals with simple obesity defined as BMI ≥ 32 kg/m^2^) and the extremely thin group (comprising lean individuals with BMI ≤ 15 kg/m^2^). Participants with secondary obesity, autoimmune diseases, familial genetic diseases, hematologic diseases, various malignant tumors, recent severe infections, major trauma, or surgery within the last 3 months prior to enrollment were excluded.

### Sample collection

A total of 2 mL venous blood samples were collected from each participant. The blood collection tubes were anticoagulated with EDTA. The blood samples were then centrifuged at 2000 rpm/min for 5 min to separate the plasma and red blood cells. These components were subsequently stored at -80 ℃ in a refrigerator. The plasma samples were used for microarray screening (miRNA screening), while the remaining plasma was reserved for PCR validation.

### miRNA microarray assay

Total RNA was extracted and purified using MirVanaTM PARISTM (Cat#AM1556, Ambion, Austin, TX, US) following the manufacturer’s instructions. The RNA samples were assessed for RNA integrity with an Agilent Bioanalyzer 2100 (Agilent Technologies, Santa Clara, CA, US) [6]. Three samples exhibited severe RNA degradation during quality assessment and were subsequently excluded. The remaining 27 samples were selected for further analysis. RNA samples were then sent to Shanghai Biotechnology Corporation (Shanghai, China) for analysis. The expression profiles of miRNAs, including 2006 mature human miRNAs, were assessed through miRNA microarray analysis using Agilent Human miRNA Array V19.0 (Agilent Technologies, Santa Clara, CA, USA). Differentially expressed miRNAs were identified using the Mann-Whitney test, with a significance threshold set at P < 0.05.

### Quantitative polymerase chain reaction (qPCR)

qPCR was conducted using an ABI 7900 HT sequence detection system (Applied Biosystems; Thermo Fisher Scientific, Inc.). Plasma levels of has-miR-126-3p, has-miR-15b-5p, has-miR-199a-3p, has-miR-20a-5p, has-miR-223-3p, has-miR-24-3p, and has-miR-23a-3p were assessed using the TaqMan microRNA reverse transcription kit (ABI, USA). To serve as an internal standard gene [7] with relatively stable expression levels, has-miR-1228-3p was chosen. The probe sequences were as follows: hsa-miR-24-3p: UGGCUCAGUUCAGCAGGAACAG hsa-miR-223-3p: UGUCAGUUUGUCAAAUACCCCA hsa-miR-20a-5p: UAAAGUGCUUAUAGUGCAGGUAG hsa-miR-199a-3a-3p: ACAGUAGUCUGCACAUUGGUUA hsa-miR-15b-5p: UAGCAGCACAUCAUGGUUUACA hsa-miR-126-3p: UCGUACCGUGAGUAAUAAUGCG hsa-miR-1228-3p: UCACACCUGCCUCGCCCCCC. The qPCR thermocycling conditions were as follows: 50 ℃ for 60 s, 95 ℃ for 10 min, followed by 40 cycles of 95 ℃ for 15 s and 60 ℃ for 60 s. The data were processed using the relative quantification method, and significant differences between groups were determined using two-tailed Student’s t-tests. Relative values were calculated using the ^2^-ΔΔCT (Livak) method [8].

### Identification of predicted miRNA target genes and functional analysis

The Mirwalk (http://mirwalk.umm.uni-heidelberg.de/) algorithm was utilized for predicting target genes of the differentially expressed miRNAs. Mirwalk is an early software for predicting miRNA target genes. In *Nature Methods*, the first version was released in 2011, followed by the V2 version in 2015, and then the V3 version at present [9]. The 1968 predicted target genes were analyzed using the Database for Annotation, Visualization, and Integrated Discovery software. Functional enrichment results were visualized through a functional network map plugin for Cytoscape, enabling visualization and comparison of functional enrichment.

### Data analysis and statistics

**Fig. 1:**
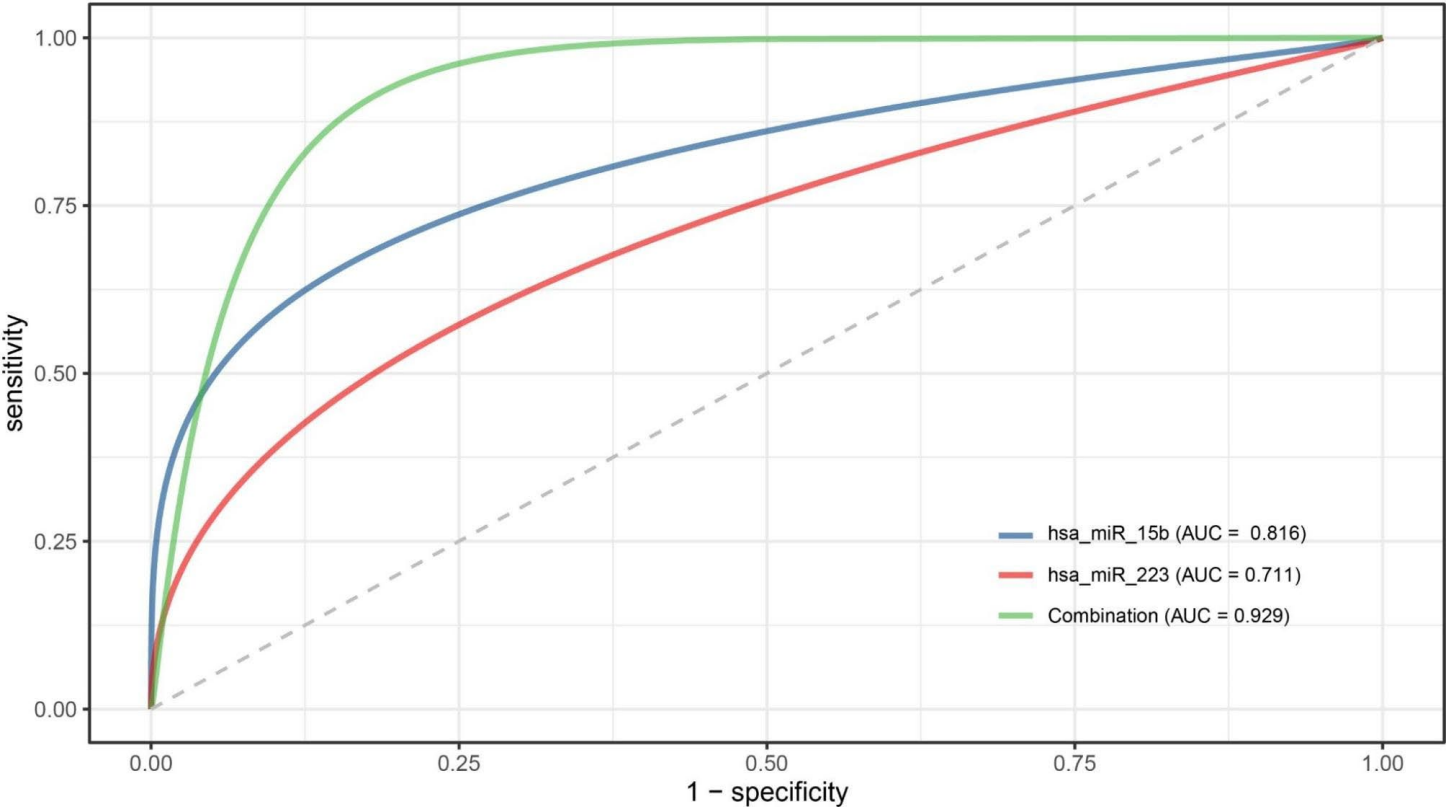
The performance of microRNAs in predicting obesity development. The assessment of the Area under the Receiver Operator Characteristic Curves (AUC) for miRNA-15b-5p, miR-223-3p, and the combination of miRNA-15b-5p and miRNA-223-3p was carried out to evaluate their predictive performance for obesity development

Microarray expression data were standardized using the internal reference gene (has-miR-1228-3p) and log2-transformed for subsequent analysis. Data were presented as mean ± SD following a normal distribution. Independent sample t-tests were applied for group comparisons. The normality of the data was assessed using the Shapiro–Wilk test method (Supplementary Table 1). Pearson correlation coefficients were calculated to analyze the correlation between clinical features and serum miRNAs. Multiple linear regression was employed to assess the correlation between miRNA expression and obesity risk. Data analysis was performed using SPSS 26.0 (SPSS, Inc., Chicago, IL, USA). The receiver operator characteristic (ROC) curve’s area under the curve (AUC) was determined to evaluate the selected miRNAs’ performance (Fig. 1). Group differences were evaluated using two-tailed Student’s t-tests, with statistical significance defined at a P-value < 0.05.

## Results

### Participant characteristics

The statistical analysis of age, sex, BMI, and FMP between the two groups was conducted. No significant differences were found in age and sex between the two groups. However, there were significant differences in BMI and FMP (Table 1).

**Table 1:** Participant Characteristics

	Lean ( N = 13 )	Obese ( N = 14 )	*p* ^a^
Age (year)	10.39 ± 0.96	12.15 ± 2.6	0.029
Sex (boy/girl)	7/6	7/7	0.842
BMI (kg/m^2^)	14.24 ± 0.62	38.94 ± 5.09	< 0.001
FMP (%)	9.42 ± 1.69	48.99 ± 2.71	< 0.001

Data are presented as the mean ± SD.

Abbreviations: BMI, body mass index; FMP, fat mass percentage; ^a^, Student t-test

### MiRNA expression profiling in obesity

Differential expression analysis identified 34 miRNAs with significant differences (P < 0.05, fold change ≥ 2, and mean = 3). After further screening using an internal reference, 36 miRNAs with significant differences in expression (P < 0.05, fold change ≥ 2, and mean = 3) were selected. Ten differentially expressed miRNAs were chosen for further investigation after intersection analysis (Table 2). Seven up-regulated miRNAs were selected for subsequent validation. The raw data from the microarray assay were normalized using the median of hsa-miR-1228-3p and analyzed with Gene Spring Software 12.6 (Agilent Technologies). The Mann-Whitney test was employed to determine differentially expressed miRNAs between the two groups.

**Table 2:** Differentially expressed miRNAs modified by the original difference and internal reference

Names	*P*-value	Fold change	Regulation
hsa-miR-21-5p	2.54E-08	6.7	down
hsa-miR-27a-3p	4.83E-05	8.0	down
hsa-miR-130a-3p	2.34E-07	5.7	down
hsa-miR-126-3p	4.13E-08	8.3	up
hsa-miR-15b-5p	2.32E-08	8.9	up
hsa-miR-199a-3p	3.44E-06	13.2	up
hsa-miR-20a-5p	0.000158	7.3	up
hsa-miR-223-3p	2.35E-07	8.4	up
hsa-miR-23a-3p	1.57E-08	8.1	up
hsa-miR-24-3p	2.36E-07	7.1	up

The Mann-Whitney test was used to determine the differentially expressed miRNAs (*P* < 0.05, fold change > 2.0, mean = 3) between obesity and lean

### Level of differential expression of miRNAs

**Fig. 2:**
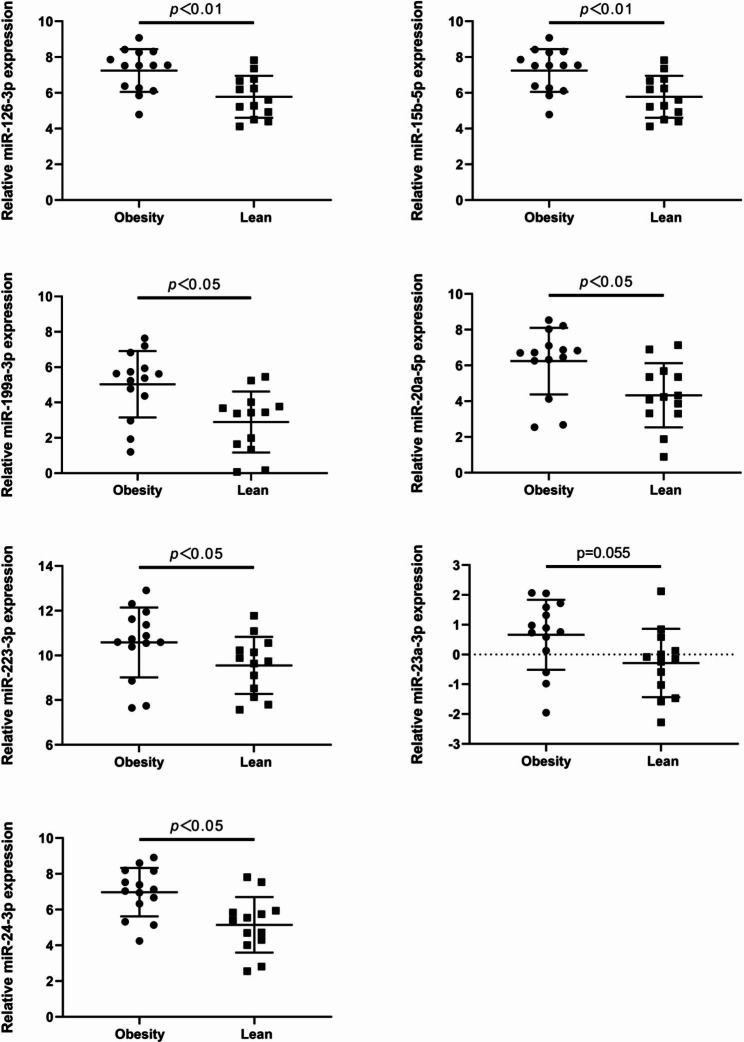
Expression of miRNAs as detected by TaqMan probes. A comparison of circulating miRNA expression profiles in lean and obese participants. The mean level with the interquartile range is displayed in the graphs. Significant differences between groups were determined using a one-sided t-test and Wilcoxon rank test. All error bars indicate mean ± SEM

The reliability of the microarray results was verified by detecting the differential miRNA expression levels using TaqMan probes. The results demonstrated significant differences in hsa-miR-126-3p, hsa-miR-15b-5p, hsa-miR-199a-3p, hsa-miR-20a-5p, hsa-miR-223-3p, and hsa-miR-24-3p between the obese and lean groups. No significant difference was observed in hsa-miR-23a-3p between the two groups (Fig. 2). The Shapiro–Wilk test method was employed to assess the normality of the data, indicating that the differentially expressed miRNAs largely had p-values greater than 0.05 (P > 0.05), indicating a generally normal distribution (Supplementary Table 1). For the TaqMan probe qPCR data, the two-tailed Student’s t-test was used to identify differentially expressed miRNAs (P < 0.05, fold change > 2.0) between the two groups.

### Correlation analysis of miRNAs expression level

The correlation between BMI, FMP, age, and hsa-miR-126-3p, hsa-miR-15b-5p, hsa-miR-199a-3p, hsa-miR-20a-5p, hsa-miR-223-3p, hsa-miR-23a-3p, and hsa-miR-24-3p was analyzed (Table 3). The results revealed positive correlations between the expression levels of hsa-miR-126-3p, hsa-miR-15b-5p, hsa-miR-199a-3p, hsa-miR-20a-5p, and hsa-miR-24-3p with BMI and FMP, while no correlations were observed with age. No significant correlations were found between hsa-miR-223-3p and BMI, FMP, or age.

**Table 3:** Correlation Analysis between miRNA and Obesity Metrics (BMI, FMP, and Age)

		hsa-miR-126-3p	hsa-miR-15b-5p	hsa-miR-199a-3p	hsa-miR-20a-5p	hsa-miR-223-3p	hsa-miR-24-3p
Age	r	0.052	0.193	0.144	0.146	0.109	0.083
	*p*	0.797	0.336	0.475	0.468	0.589	0.681
BMI	r	0.493^**^	0.550^**^	0.520^**^	0.506^**^	0.370	0.520^**^
	*p*	0.009	0.003	0.005	0.007	0.058	0.005
FMP	r	0.534^**^	0.518^**^	0.515^**^	0.472^*^	0.348	0.534^**^
	*p*	0.004	0.006	0.006	0.013	0.075	0.004

Note: * is the rank correlation coefficient, *P* < 0.05, and * * is the rank correlation coefficient, *P* < 0.01

Abbreviations: r, Pearson’s correlation analysis; *p*, *p*-value

### Association between miRNA and obesity risk

The correlation between miRNA expression and obesity risk was assessed using multiple linear regression. Two models were employed, and the results indicated significant impacts in both models. In model one, hsa-miR-15b-5p (β = 10.529, p = 0.001) and hsa-miR-223-3p (β =-10.225, p = 0.010) exhibited significant associations with obesity. Higher expression levels of hsa-miR-15b-5p and hsa-miR-223-3p were strongly correlated with obesity. In model two, even after adjusting for sex and age as confounding factors, the association between these two miRNAs and obesity remained significant (hsa-miR-15b-5p: β = 8.936, p = 0.002; hsa-miR-223-3p: β =-8.499, p = 0.022) (Table 4). ROC curve analysis was performed to assess the performance of selected miRNAs (Fig. 1). The AUC values were calculated with logistic regression and reached 0.816, 0.711, and 0.929 for hsa-miR-15b-5p, hsa-miR-223-3p, and the combined miRNA classifiers (hsa-miR-15b-5p + hsa-miR-223-3p), respectively. These results indicate that these miRNAs have the ability to effectively distinguish between obese and lean individuals with relatively high sensitivity and specificity. Additionally, odds ratio analysis was conducted to evaluate the relationship between miRNA expression and obesity in children (Table 5). After adjusting for age and sex, a significant correlation was observed between hsa-miR-15b-5p and obesity (OR = 143, 95% CI 5.80 to 56,313, p = 0.024), as well as between hsa-miR-223-3p and obesity (OR = 0.01, 95% CI 0.00 to 0.23, p = 0.037). These findings suggest that hsa-miR-15b-5p and hsa-miR-223-3p are strong predictive candidates for distinguishing between healthy individuals and those with obesity.

**Table 4:** Multiple Linear Regression Analysis of miRNA and Obesity in Children

miRNA	Model 1				Model 2		
	*β*	95%*CI*	*P*-value^*^		*β*	95%*CI*	*P*-value^*^
hsa-miR-15b-5p	10.529	4.974~16.084	0.001		8.936	3.572~14.301	0.002
hsa-miR-223-3p	-10.225	-17.852~-2.657	0.010		-8.499	-15.634~-1.303	0.022

Note: Independent variables of model 1 were miRNAs hsa-miR-15b-5p and hsa-miR-223-3p; independent variables of model 2 were miRNAs hsa-miR-15b-5p and hsa-miR-223-3p and adjustment factors age and gender. It still had significance after multiple tests after correction; ^*^P < 0.05. Multiple linear regression was used for correlation analysis between miRNA expression and obesity risk

**Table 5:** Risk Assessment for the Association between miRNA and Childhood Obesity

Characteristic	OR^1^	95% CI^1^	*P*-value
has-miR-15b-5p	143	5.80~56,313	0.024
has-miR-223-3p	0.01	0.00~0.23	0.037

^1^OR = Odds Ratio, CI = Confidence Interval

Multivariate logistic regression analysis was conducted

### Pathway analysis for predicted targets of miRNAs

The miRWalk database was utilized to predict potential target genes of hsa-miR-15b-5p and hsa-miR-223-3p. A total of 1968 predicted target genes were identified for the two selected miRNAs. Among these, hsa-miR-15b-5p exhibited a substantial number of target genes associated with obesity-related functions. The target genes of hsa-miR-15b-5p were involved in several pathways, including the FoxO signaling pathway, insulin signaling pathway, Ras signaling pathway, AMPK signaling pathway, endocrine and other factor-regulated calcium reabsorption, cAMP signaling pathway, prolactin signaling pathway, signaling pathways regulating pluripotency of stem cells, sphingolipid signaling pathway, and thyroid hormone signaling pathway.

**Fig. 3:**
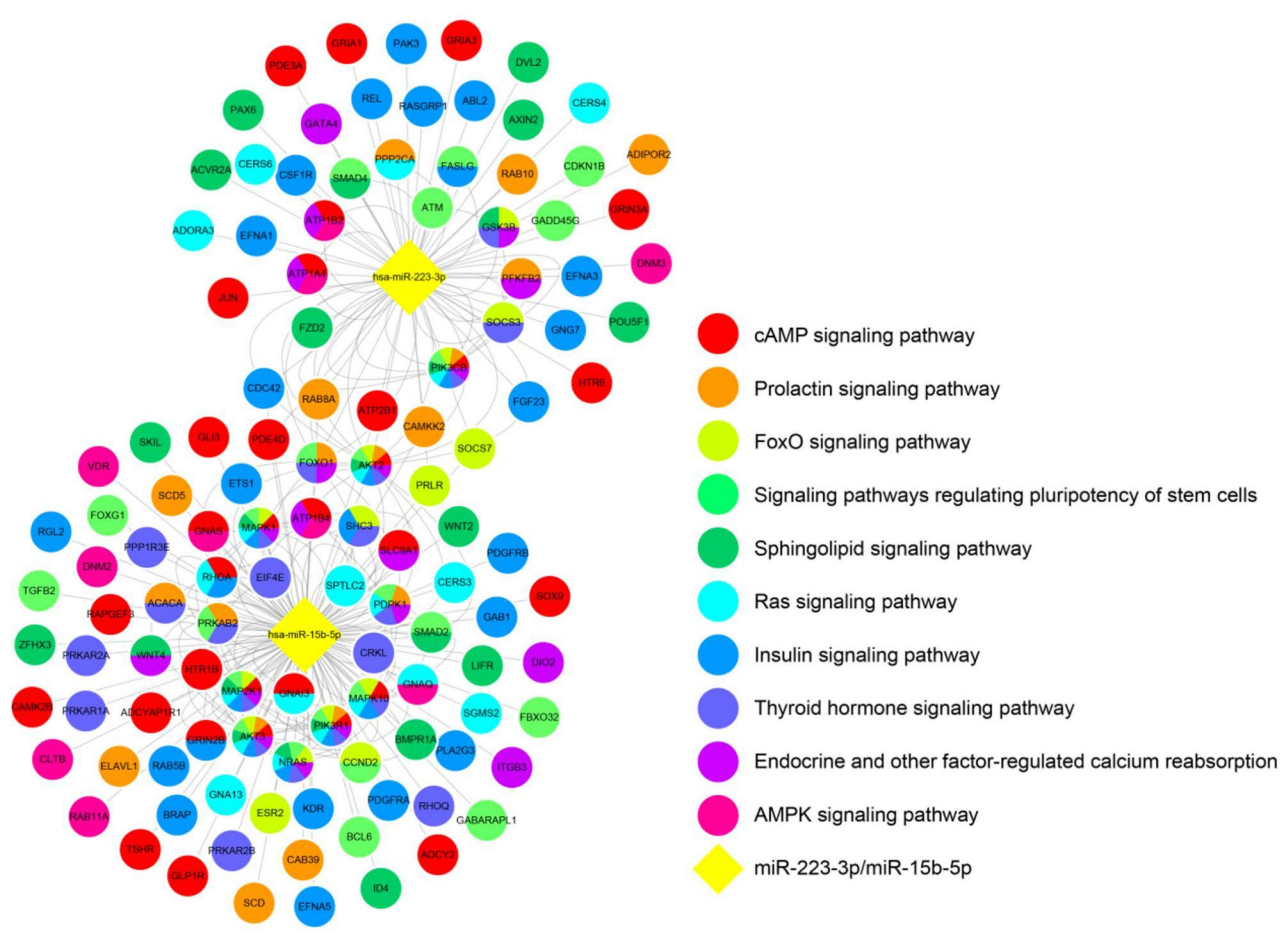
Mapping of target genes for miRNA-15b-5p and miR-223-3p, which were significantly upregulated in obese participants. The landscape of genes associated with biological processes was visualized using Cytoscape software. Each miRNA was represented by a diamond node, and target mRNAs were represented by circular nodes. The pathway involved by the target genes was indicated by the node color

Figure 3 illustrates the pathway network for the predicted targets of these two miRNAs, highlighting their association with various obesity-related pathways, such as FoxO, insulin, Ras, and AMPK signaling pathways. Each node in the network represents a group of genes, with diamond nodes representing miRNAs and circular nodes representing mRNAs. Node color indicates the fold changes in miRNA expression, with yellow indicating upregulation.

## Discussion

Metabolic diseases, including type 2 diabetes mellitus, dyslipidemia, atherosclerosis, hypertension, and diabetes, exhibit a positive correlation with the increasing prevalence of obesity. Epidemiological surveys have revealed that over the past three decades, approximately 35% of adults and 20% of children in the United States and other nations have developed obesity [10]. Importantly, the risk factors associated with obesity, such as hypertension, coronary heart disease, diabetes, and metabolic syndrome, independently contribute to the risk of coronary heart disease and ischemic stroke. Effective weight control is a pivotal strategy for both the prevention and treatment of chronic diseases.

Recent years have witnessed a wealth of evidence highlighting the crucial role of miRNAs in the epigenetic regulation of adipogenesis [11, 12] and obesity [13, 14]. A previous study [15] emphasized the significance of miRNAs in fat development and overall body functioning. Therefore, unraveling the mechanisms governing miRNAs is of paramount importance. Circulating microRNAs, as emerging endocrine factors, can be released from various tissues and act as endocrine and paracrine messengers, facilitating communication between donor cells, recipient cells, or target tissues [16]. A growing body of research indicates that circulating miRNAs are closely associated with obesity in adults, young individuals, and children [17, 18]. For instance, a study involving preschool obese children aged 2–6 years found that circulating miR-190a and miR-95 could serve as molecular markers for insulin resistance in obese children [19]. Another study involving 70 children aged 5–10 years suggested that plasma miRNAs might serve as a potential screening tool for identifying endothelial dysfunction in children with cardiovascular diseases [20]. Furthermore, Ortega [21] conducted an analysis of the relationship between various levels of circulating miRNA expression and obesity, as well as weight loss in adults. Carlos discovered that obesity alters the miRNA profile of plasma exosomes in mice, leading to increased expression levels of miR-122, miR-192, miR-27a-3p, and miR-27b-3p. Treating lean mice with exosomes isolated from obese mice resulted in glucose intolerance and insulin resistance [22]. In our study, we observed significant differences in the expression levels of miRNAs in the circulating plasma of obese and lean children aged 8–12 years. To effectively highlight the phenotypic distinctions between these two groups, we deliberately selected participants with extreme BMIs, specifically those with simple obesity (BMI ≥ 32 kg/m^2^) and those classified as lean (BMI ≤ 15 kg/m^2^). This strategic choice of two groups situated at opposite ends of the BMI spectrum allowed us to enhance the statistical power of our study, making it more sensitive to the identification of miRNAs with larger effect sizes. Among the miRNAs analyzed, hsa-miR-126-3p, hsa-miR-15b-5p, hsa-miR-199a-3p, hsa-miR-20a-5p, hsa-miR-223-3p, hsa-miR-23a-3p, and hsa-miR-24-3p exhibited upregulated expression in the obese group, while hsa-miR-21-5p, hsa-miR-27a-3p, and hsa-miR-130a-3p displayed downregulated expression. These observed alterations in miRNA expression have the potential to influence the body’s growth and development through their evident regulatory roles, thereby playing a pivotal role in the context of obesity. Numerous studies have indicated associations between circulating miRNAs, such as miR-15b-5p, miR-486-5p, and miR-122-5p, and obesity and overweight conditions. An analysis of four high-throughput sequencing studies has suggested that these miRNAs hold promise as potential biomarkers for obesity [23]. Furthermore, miR-486, miR-146b, and miR-15b have been found to be significantly expressed in the circulation of both obese children and adults with type 2 diabetes. Among these, miR-486 is involved in promoting pre-adipocyte proliferation and enhancing myotube glucose tolerance. Meanwhile, miR-146b and miR-15b play roles in inhibiting pancreatic insulin secretion induced by high glucose concentrations, contributing to the pathological processes of obesity and type 2 diabetes [24]. In a separate study, 16 out of the 20 miRNAs tested in obese children exhibited expression levels that were twice as high as those in the control group. Notably, miR-199 and miR-122 were remarkably elevated in obese children [25]. Adipogenesis represents a pluripotent differentiation process wherein mesenchymal stem cells can differentiate into adipocytes via adipocyte precursor cells [26]. The influence of miRNAs on adipocyte differentiation is multifaceted, with some miRNAs promoting the process while others inhibit it [27]. For example, miR-143 was the first miRNA identified in relation to adipocyte differentiation, with its expression gradually increasing as human adipose precursor cells differentiate and mature [28]. Price’s research showed that miR-33b overexpression can inhibit pre-adipocyte proliferation and reduce lipid droplet formation during adipocyte differentiation [29]. In both human abdominal adipose tissue and obese mice, the high expression of miR-146a has been shown to reduce the inflammatory response of adipocytes by inhibiting the JNK and p38 signaling pathways [30]. In conclusion, the precise mechanisms by which miRNAs regulate adipocyte differentiation warrant further investigation.

The results of the TaqMan probe qPCR experiment indicated a positive correlation between the expression levels of hsa-miR-126-3p, hsa-miR-15b-5p, hsa-miR-199a-3p, hsa-miR-20a-5p, and hsa-miR-24-3p with BMI and FMP, although there was no significant correlation with age, except for hsa-miR-23a-3p. This suggests that these six miRNAs could potentially serve as markers of obesity. Multiple linear regression analysis and ROC curve analysis both supported a correlation between miRNA expression and obesity. Furthermore, fold risk analysis predicted a high-risk association between miRNA expression and obese children. Notably, the expression levels of hsa-miR-15b-5p and hsa-miR-223-3p remained significantly different between the obese and lean groups, even after adjusting for age and sex. However, the specific mechanisms underlying these associations require further investigation.

Pathway analysis revealed that hsa-miR-15b-5p and hsa-miR-223-3p are involved in numerous obesity-related functions, including the FoxO, insulin, Ras, and AMPK signaling pathways. Of particular interest, hsa-miR-15b-5p-regulated genes were associated with multiple functions. Taken together, the altered expression of hsa-miR-15b-5p and hsa-miR-223-3p in obese individuals may influence the pathophysiology of obesity, insulin resistance, and diabetes.

This study aimed to pinpoint the crucial miRNAs governing obesity through microarray and correlation analyses. The results indicate that hsa-miR-15b-5p and hsa-miR-223-3p hold promise as early intervention targets for childhood obesity. However, it is necessary to increase the sample size for further research. In addition, the related mechanisms must be elaborated at the cellular and animal levels.

## Conclusions

In conclusion, this study sought to identify key miRNAs influencing obesity using microarray and correlation analyses. Hsa-miR-15b-5p and hsa-miR-223-3p were linked to obesity, even after adjusting for age and sex. The findings suggest that hsa-miR-15b-5p and hsa-miR-223 have more predicted target genes related to obesity than other miRNAs. Specifically, hsa-miR-15b-5p has numerous target genes associated with the FoxO, insulin, Ras, and AMPK signaling pathways. Hsa-miR-15b-5p and hsa-miR-223-3p show promise as early intervention targets for childhood obesity.

### Electronic supplementary material

Below is the link to the electronic supplementary material.

**Supplementary Material 1: Supplementary Table 1**. Statistical Summary and Normality Tests for Data

## Data Availability

The data presented in this study are available in article. Data cited in this article have been uploaded https://www.ncbi.nlm.nih.gov/geo/query/acc.cgi?acc=GSE227788 GEO Submission (GSE227788).

## List of abbreviations

AMPK: Adenosine 5‘-monophosphate (AMP)-activated protein kinase
AUC: Area under the ROC curve
BMI: Body mass index
cAMP: Cyclic Advenosine Monophosphato
FMP: Fat mass percentage
FoxO1: Forkhead box-containing protein, O subfamily 1
Has-miR: Homo sapiens-microRNA
JNK: C-Jun N-terminal kinase
OR: Odd ratio
qPCR: Quantitative polymerase chain reaction
ROC: The Receiver operator characteristic

## References

[1] Kumar S, Kaufman T (2018). Childhood obesity. Panminerva Med.

[2] Kumar S, Kelly AS (2017). Review of childhood obesity: from epidemiology, etiology, and comorbidities to Clinical Assessment and Treatment. Mayo Clin Proc.

[3] Ji CY, Cheng TO (2009). Epidemic increase in overweight and obesity in Chinese children from 1985 to 2005. Int J Cardiol.

[4] Flodmark CE, Lissau I, Pietrobelli A (2005). Child and adolescent obesity: why we need to fight!. Acta Paediatr (Oslo Norway: 1992) Supplement.

[5] Research Group of Chinese Students Physique and Health (2002). Report on the physical fitness and health surveillance of Chinese school students.

[6] Zhou J, Yu L, Gao X, Hu J, Wang J, Dai Z (2011). Plasma microRNA panel to diagnose Hepatitis B virus-related hepatocellular carcinoma. J Clin Oncology: Official J Am Soc Clin Oncol.

[7] Sticht C, De La Torre C, Parveen A, Gretz N (2018). miRWalk: an online resource for prediction of microRNA binding sites. PLoS ONE.

[8] Flegal KM, Carroll MD, Ogden CL, Curtin LR (2010). Prevalence and trends in obesity among US adults, 1999–2008. JAMA.

[9] McGregor RA, Choi MS (2011). microRNAs in the regulation of adipogenesis and obesity. Curr Mol Med.

[10] Alexander R, Lodish H, Sun L (2011). MicroRNAs in adipogenesis and as therapeutic targets for obesity. Expert Opin Ther Targets.

[11] Hilton C, Neville MJ, Karpe F (2013). MicroRNAs in adipose tissue: their role in adipogenesis and obesity. Int J Obes.

[12] Wang R, Hong J, Cao Y, Shi J, Gu W, Ning G (2015). Elevated circulating microRNA-122 is associated with obesity and insulin resistance in young adults. Eur J Endocrinol.

[13] Mudhasani R, Puri V, Hoover K, Czech MP, Imbalzano AN, Jones SN (2011). Dicer is required for the formation of white but not brown adipose tissue. J Cell Physiol.

[14] Ji C, Guo X (2019). The clinical potential of circulating microRNAs in obesity. Nat Reviews Endocrinol.

[15] Arner P, Kulyté A (2015). MicroRNA regulatory networks in human adipose tissue and obesity. Nat Reviews Endocrinol.

[16] Deiuliis JA (2016). MicroRNAs as regulators of metabolic Disease: pathophysiologic significance and emerging role as biomarkers and therapeutics. Int J Obes.

[17] Masotti A, Baldassarre A, Fabrizi M, Olivero G, Loreti MC, Giammaria P (2017). Oral glucose tolerance test unravels circulating miRNAs associated with insulin resistance in obese preschoolers. Pediatr Obes.

[18] Khalyfa A, Kheirandish-Gozal L, Bhattacharjee R, Khalyfa AA, Gozal D (2016). Circulating microRNAs as potential biomarkers of endothelial dysfunction in obese children. Chest.

[19] Ortega FJ, Mercader JM, Catalán V, Moreno-Navarrete JM, Pueyo N, Sabater M (2013). Targeting the circulating microRNA signature of obesity. Clin Chem.

[20] Castaño C, Kalko S, Novials A, Párrizas M (2018). Obesity-associated exosomal miRNAs modulate glucose and lipid metabolism in mice. Proc Natl Acad Sci USA.

[21] Flórez CAR, García-Perdomo HA, Escudero MM (2020). MicroRNAs Associated with overweight and obesity in Childhood: a systematic review. MicroRNA (Shariqah United Arab Emirates).

[22] Cui X, You L, Zhu L, Wang X, Zhou Y, Li Y (2018). Change in circulating microRNA profile of obese children indicates future risk of adult Diabetes. Metab Clin Exp.

[23] Thompson MD, Cismowski MJ, Serpico M, Pusateri A, Brigstock DR (2017). Elevation of circulating microRNA levels in obese children compared to healthy controls. Clin Obes.

[24] Heinonen S, Saarinen L, Naukkarinen J, Rodríguez A, Frühbeck G, Hakkarainen A et al. Adipocyte morphology and implications for metabolic derangements in acquired obesity. International journal of obesity (2005). 2014;38(11):1423-31. 10.1038/ijo.2014.31 24549139

[25] Klöting N, Berthold S, Kovacs P, Schön MR, Fasshauer M, Ruschke K (2009). MicroRNA expression in human omental and subcutaneous adipose tissue. PLoS ONE.

[26] Esau C, Kang X, Peralta E, Hanson E, Marcusson EG, Ravichandran LV (2004). MicroRNA-143 regulates adipocyte differentiation. J Biol Chem.

[27] Price NL, Holtrup B, Kwei SL, Wabitsch M, Rodeheffer M, Bianchini L (2016). SREBP-1c/MicroRNA 33b genomic loci control adipocyte differentiation. Mol Cell Biol.

[28] Roos J, Enlund E, Funcke JB, Tews D, Holzmann K, Debatin KM (2016). miR-146a-mediated suppression of the inflammatory response in human adipocytes. Sci Rep.

[29] Keller A, Leidinger P, Meese E, Haas J, Backes C, Rasche L (2015). Next-generation sequencing identifies altered whole blood microRNAs in neuromyelitis optica spectrum disorder which may permit discrimination from multiple sclerosis. J Neuroinflamm.

[30] Livak KJ, Schmittgen TD. Analysis of relative gene expression data using real-time quantitative PCR and the 2(-Delta Delta C(T)) Method. Methods (San Diego, Calif). 2001;25(4):402-8. 10.1006/meth.2001.1262 11846609

