# Supplementary material for: Identification of novel circulating miRNAs biomarkers for healthy obese and lean children
Source: BMC Endocr Disord. 2023 Oct 30;23:238. doi: 10.1186/s12902-023-01498-w (PMC10614305; doi:10.1186/s12902-023-01498-w)
Supplement: Supplementary file 1 — Supplementary Material 1: Supplementary Table 1. Statistical Summary and Normality Tests for Data [file 12902_2023_1498_MOESM1_ESM.docx]

Supplementary table 1. Statistical Summary and Normality Tests for Data.

| group | | Shapiro-Wilk test | | |
| --- | --- | --- | --- | --- |
|  |  | statistic | degree | *P*-value |
| hsa-miR-126 | Lean | 0.961 | 13 | 0.771 |
|  | Obesity | 0.945 | 14 | 0.485 |
| hsa-miR-15b-5p | Lean | 0.932 | 13 | 0.365 |
|  | Obesity | 0.859 | 14 | 0.030 |
| hsa-miR-199a-3p | Lean | 0.932 | 13 | 0.357 |
|  | Obesity | 0.923 | 14 | 0.243 |
| hsa-miR-20a | Lean | 0.968 | 13 | 0.874 |
|  | Obesity | 0.849 | 14 | 0.021 |
| hsa-miR-223 | Lean | 0.968 | 13 | 0.872 |
|  | Obesity | 0.913 | 14 | 0.176 |
| hsa-miR-23a-3p | Lean | 0.975 | 13 | 0.946 |
|  | Obesity | 0.922 | 14 | 0.236 |
| hsa-miR-24 | Lean | 0.957 | 13 | 0.710 |
|  | Obesity | 0.956 | 14 | 0.656 |
